# Supplementary material for: Thalattosuchian crocodylomorphs from European Russia, and new insights into metriorhynchid tooth serration evolution and their palaeolatitudinal distribution
Source: PeerJ. 2023 Aug 11;11:e15781. doi: 10.7717/peerj.15781 (PMC10424675; doi:10.7717/peerj.15781)
Supplement: Supplemental Information 1 — Note, some localities are listed as “NA” = not applicable, as paleolatitude.org could not compute their palaeolatitude due to the coordinates being located on an unconstrained plate. [file peerj-11-15781-s001.docx]

**Table S1.** List of major metriorhynchoid-bearing formations, and approximate coordinates. Note, some localities are listed as “NA” = not applicable, as paleolatitude.org could not compute their palaeolatitude due to the coordinates being located on an unconstrained plate.

| **Taxa** | **Stratigraphy and age** | **Locality** | **GPS coordinates and palaeolatitudes** | **References** |
| --- | --- | --- | --- | --- |
| **Chile** | | | | |
| Metriorhynchoidea indet. | Uppermost of the Lautaro Formation.  Early Bajocian. | Quebrada de la Iglesia, Atacama | 28.17 S, 69.88 W  (27.31–36.54S for 170 Ma) | Gasparini *et al*., 2000; Pol & Gasparini, 2009; Soto-Acuña & Otero, 2014; Soto-Acuña *et al*., 2015a |
| “*Metriorhynchus*” *westermanni* | Quehuita Member, Quehuita Formation.  Early Callovian. | Quebrada Cherejara, Sierra del Medio, Antofagasta | 24.166 S, 68.667 W  (27–26S for 170–160 Ma) | Gasparini *et al*., 2008; Soto-Acuña *et al*., 2015a |
| “*Metriorhynchus*” *westermanni* | Mina Chica Fm., early-middle Callovian | Placilla de Caracoles, Antofagasta | 23.036S, 69.005 W  (26–25S for 170–160 Ma) | Gasparini, 1980; Soto- Acuña *et al*., 2015a |
| “*Metriorhynchus*” *casamiquelai* | Lower Member, Quinchamale Formation.  Middle Callovian | Quebrada Sajasa, Sierra Moreno, Antofagasta  Sierra Candeleros, Antofagasta | 21.875 S,  69.116 W  (25–24S for 170–160 Ma)  25.382 S, 69.368 W  (29–28S for 170–160 Ma) | Gasparini & Chong Diaz, 1977; Soto-Acuña *et al*., 2015a |
| “*Metriorhynchus*” aff. *westermanni* | beds with ‘*Perisphinctes*’ *andium*, Sierra El Cobre Formation. Oxfordian. | Cerro Laberinto, Cordillera de Domeyko, Antofagasta | 23.440 S, 69.344 W  (31–21 S (25.3 S for 160 Ma) | Fernández *et al*., 2011 |
| Metriorhynchidae indet. | beds with ‘*Perisphinctes*’ *andium*, Cerritos Bayos Formation. Oxfordian. | Cerritos Bayos, Calama, Antofagasta. | 22.617 S; 69.167 W  (30–20S (25)S for 160 Ma) | Soto-Acuña *et al*., 2015a, 2015b |
| ?*G*. *araucanensis*  Thalattosuchia indet. (Soto-Acuña *et al*., 2015a) | Lo Valdés Formation.  Tithonian | Lo Valdéz, Chile | 33.823 S, 70.052 W  (35–30S for 150–140 Ma) | Gasparini, 1985; Pol & Gasparini, 2009; Soto-Acuña *et al*., 2015a |
| **Argentina** | | | | |
| a thalattosuchian vertebra | Los Molles Formation.  Early Bajocian. | Chacaico Sur, southwest of the Neuquén Basin | 39.446 S, 70.305 W  (47.77–38.55S 42.93S for 170 Ma) | Pol & Gasparini, 2009 |
| *Metriorhynchus* aff. *M*. *brachyrhynchus*  Geosaurinae indet. | Middle to upper section of the Los Molles Formation.  Upper Bathonian | Chacay Melehue, located 27 km norrhwest of Chos Malal, Neuquén Province, Argentina | 37.224 S, 70.369 W  (41–40S for 170–160 Ma) | Gasparini *et al*., 2005; Pol & Gasparini, 2009 |
| *Cricosaurus araucanensis* | Portada Covunco Member.  Middle Tithonian. | Cerro Lotena and Bosque Petrificado (about 7 km to southwest of Cerro Lotena)  Yesera del Tromen-Pampa Tril (Tithonian-Berriasian).  Tithonian levels of Sierra de Reyes in Mendoza Province | 39.184 S, 69.654 W  (40–35S for 150–140 Ma)  37.238 S, 69.810 W  (38–33S for 150–140 Ma) | Gasparini & Dellapé, 1976; Herrera, 2015 |
| *Cricosaurus* *lithographicus* | *Aulacosphinctes proximus*–*Windhauseniceras intenispinosum* Biozones, Los Catutos Member, Vaca Muerta Formation.  Upper lower–middle upper Tithonian. | El Ministerio quarry, Los Catutos Area, Neuquén Province | 38.853 S, 70.182 W  (40–35S for 150–140 Ma) | Herrera, Gasparini & Fernández, 2013; Herrera, 2015 |
| *Dakosaurus* *andiniensis* Vignaud & Gasparini, 1996 | Vaca Muerta Formation.  Tithonian–Berriasian  Levels. | Catan Lil, Barrancas River (Malargüe, Mendoza Province)  in Berriasian of Mallín Quemado area (Neuquén Province)  Tithonian of Yesera del Tromen-Pampa Tril area (Neuquén Province) | 36.481 S, 70.178 W  (37,5–32S for 150–140 Ma)  38.583 S, 70.083 W  (40–34S for 150–140 Ma)  37.238 S, 69.810 W (38–33S for 150–140 Ma) | Vignaud & Gasparini, 1996; Gasparini *et al*., 2006 |
| *Dakosaurus* cf. *D*. *andiniensis* | *Substeueroceras koeneni* Biozone, Vaca Muerta Formation.  Uppermost Tithonian to lowermost Berriasian. | Vega de Escalone locality (Neuquén Province) | 37.185 S, 69.795 W  (38–33S for 150–140 Ma) | Herrera, 2015 |
| *Purranisaurus* *potens* Rusconi, 1948a | *Substeueroceras koeneni* Biozone, Vaca Muerta Formation.  Upper Tithonian–lower Berriasian. | Arroyo del Cajón Grande, southwest of Malargüe Department, Mendoza Province | 35.894 S, 70.314 W  (37–32S for 150–140 Ma) | Herrera, 2015; Herrera *et al*., 2015 |
| Metriorhynchidae indet. and Geosaurini indet.; *Cricosaurus* sp. | Upper Tithonian, *Corongoceras alternans* Biozone; Upper Tithonian–lower Berriasian *Substeueroceras koeneni* Biozone | Arroyo Durazno and Arroyo Paulino | 35.08 S  69.75 W;  34.967 S, 69.817 W  (36–31S for 150–140 Ma) | Fernández *et al*., 2019 |
| *Cricosaurus* *puelchorum* Herrera *et al*., 2021a | lower part of the *Argentiniceras noduliferum* Biozone, Vaca Muerta Formation.  Lower Berriasian, | Arroyo Paulino cropping out on the right margin of the Paulino Creek, Mendoza Province, Argentina | 34.967 S, 69.817 W  (36–31S for 150–140 Ma) | Herrera *et al*., 2021a |
| **Colombia** | | | | |
| cf. *Cricosaurus* | *Sayonoceras verrucosum* Biozone, Rosa Blanca Formation.  Upper Valanginian. | Colombia | NA. overall low tropical latitudes | Larsson *et al*., 2012 |
| **Mexico** | | | | |
| Thalattosuchia indet.,  Metriorhynchidae indet. | Tecocoyunca group.  Callovian. | Cualác, Guerrero, Mexico | 17.788N 98.645W  (7–10N for 170–160 Ma) | Gasparini, 1992; Cadena-Rangel, 2015 |
| *Maledictosuchus* *nuyivijanan* Barrientos-Lara, Alvarado-Ortega & Fernández, 2018 | Sabinal Formation. Kimmeridgian | Llano Yosobe, near Tlaxiaco, Oaxaca, southern Mexico | 17.247 N, 97.695 W  (9–13N for 160–150 Ma) | Barrientos-Lara *et al*., 2018 |
| *Torvoneustes* *mexicanus* | Sabinal Formation. ?Kimmeridgian | 6 km to south-southwest from Tlaxiaco town, Oaxaca | ~ 17.214N, 97.679W  (9–13N for 160–150 Ma) | Wieland, 1910; Buchy, 2008a; Barrientos-Lara *et al*., 2016 |
| Geosaurini indet. (was *Dakosaurus* sp.) | La Casita Formation.  Kimmeridgian | San Juan de los Dolores, Coahuila | 25.431 N, 100.582W  (18–21N for 160–150 Ma) | Buchy *et al*., 2007; Buchy, 2008b; Young *et al*., 2012 |
| *‘Cricosaurus’* *saltillensis* | La Caja Formation.  Early Tithonian. | Sierra de Buñuelas, near Gómez Farías, Coahuila | 25.057N, 101.145W  (21–29N for 150–140 Ma) | Buchy *et al*., 2006, 2013 |
| *Cricosaurus* *vignaudi* | La Pimienta Formation.  Middle Tithonian. | Mazatepec, Puebla | 20.016N, 97.416W  (15–23N for 150–140 Ma) | Frey *et al*., 2002 |
| **Cuba** | | | | |
| *Cricosaurus sp.* | Oxfordian | Southern slope of the Sierra de Guasasa; Cuba | 22.653 N, 83.704 W  (NA) | Gasparini & Iturralde-Vinent, 2001, 2006; Young & Andrade, 2009 |
| Metriorhynchidae indet.; Thalattosuchia indet.; | Oxfordian | Puerta del Ancón, Sierra de Guasasa  Hoyo de la Sierra | 22.653 N, 83.704 W  22.664 N, 83.498 W  (NA) | Gasparini & Iturralde-Vinent, 2006 |
| **USA** | | | | |
| *Zoneait* *nargorum* | Weberg Member, Snowshoe Formation. Latest Aalenian or early Bajocian. | near the towns of Suplee and Izee, east-central Oregon, USA | 44.089 N, 119.392 W  (NA) | Buffetaut, 1979; Wilberg, 2015 |
| **Europe early-diverging metriorhynchoids** | | | | |
| *Pelagosaurus* *typus* | Whitby Mudstone Formation.  Early Toarcian. | Whitby area, Yorkshire, England | 54.48 N, 0.62 E  (42N for 180Ma) | e.g. Westphal, 1962; Benton & Taylor 1984; Young & Steel, 2019 |
| *Pelagosaurus* *typus* | Beacon Limestone Formation.  Early Toarcian. | Strawberry Bank, north of Ilminster, Somerset, England | 50.55 N 2.54 W  (38N for 180Ma) | e.g. Duffin, 1979; Pierce & Benton, 2006 |
| *Pelagosaurus* *typus* | Argiles à Poissons Formation.  Early Toarcian. | Curcy-sur-Orne, Normandy, France | 49.01N, 0.52W  (37N for 180Ma) | e.g. Eudes-Deslongchamps, 1864; Steel, 1973; Brignon, 2018 |
| *Pelagosaurus* *typus* | Sachrang Formation.  Early Toarcian. | Holzmaden area, Baden-Württemberg, Germany | 48.38 N, 9.31 E  (39N for 180Ma) | Westphal, 1961,  1962; Steel, 1973 |
| *Magyarosuchus fitosi* | *Grammoceras* *thouarsense* ammonite Biozone, Kisgerecse Marl Formation.  Late Toarcian | Nagy-Pisznice Hill, close to Békás-Canyon, Hungary | 47.42 N, 18.29 E  (NA) | Ősi *et al*., 2018 |
| *Opisuchus meieri* | Opalinuston Formation.  Early Aalenian. | Heiningen near Göppingen, Baden-Württemberg, Germany | 48.39 N, 9.38 E  (39–32N for 180Ma–170Ma) | Aiglstorfer *et al*., 2020 |
| ‘*Pelagosaurus’* *tomarensis* | uppermost Toarcian or the base of the Aalenian | Tomar, Portugal | 39.36 N, 8.25 W (30–25N for 180Ma–170Ma) | Ferreira, 1959; Telles Antunes, 1967 |
| *Teleidosaurus calvadosii* | *zigzag*–*progracilis* Biozones, Calcaire de Caen and Calcaire d’Ecouché Formations.  Lower–middle Bathonian. | Calvados and Ecouché, Normandy, France | 49.02 N, 0.15 E  (30N for 170Ma)  48.43 N, 0.73 W  (30N for 170Ma) | Eudes-Deslongchamps, 1866; Young *et al*., 2010; Hua, 2020 |
| *Eoneustes bathonicus*  *Eoneustes gaudryi* | Upper Bajocian–Middle Bathonian | Normandy, Burgundy, and Castellane, France.  Départements of Cote d’Or, Burgundy, and Alpes-de-Haute-Provence, France | 49.02 N, 0.15 E  (30N for 170Ma)  47.26 N, 4.47 E  (30N for 170Ma) | Mercier, 1933; Collot, 1905; Young *et al*., 2010 |
| **Europe Metriorhynchidae** | | | | |
| *Neptunidraco ammoniticus* | Rosso Ammonitico Veronese Formation. Lower-most Bathonian | Near Sant'Ambrogio di Valpolicella, Verona, Italy | 45.31 N, 10.50 E  (25N for 170Ma) | Cau & Fanti, 2011 |
| *Thalattosuchus superciliosus*  *Gracilineustes leedsi*  *‘Metriorhynchus’ brachyrhynchus*  *Tyrannoneustes lythrodectikos*  *Ieldraan melkshamensis*  *Suchodus durobrivensis* | Oxford Clay Formation. Middle Callovian. | Most specimens are known from the Peterborough area of the UK.  Some localities further south also bear metriorhynchids, such as near Melksham, Wiltshire. | 52.57 N, 0.24 W  (34–35N for 170Ma–160Ma)  51.37 N, 2.14 W  (32–34N for 170Ma–160Ma) | Andrews, 1913; Foffa *et al*., 2018; Young *et al*., 2013a, 2021 |
| *Thalattosuchus superciliosus*  *‘Metriorhynchus’ brachyrhynchus*  *Tyrannoneustes* sp. (NHMUK PV OR 47989)  *Suchodus durobrivensis* | Marnes de Dives Formation.  Upper Callovian and lower Oxfordian. | Falaises des Vaches Noire, Calvados, France | 49.18 N, 0.05W  (30–32N for 170Ma–160Ma) | Eudes-Deslongchamps, 1867-1869; Lepage *et al*., 2008; Young *et al*., 2013a |
| Metriorhynchidae indet. | lower part of the Lower Callovian | Lacha-pelle-sous-Aubenas village, Monts d’Ardèche UNESCO Global Geopark | 44.56 N, 4.37E  (27–29N for 170Ma–160Ma) | Riou *et al*., 2021 |
| cf. *Torvoneustes*  *Torvoneustes* sp.  cf. *Tyrannoneustes*  cf. *Thalattosuchus* *superciliosus*  Geosaurinae indet.  Metriorhynchidae indet. | Corallian Group.  Middle Oxfordian. | Coral Rag of Yorkshire (e.g., Malton, North Grimston), Headington, Quarry Fields, and Garsington Oxfordshire, Heddington of Wiltshire, UK | 54.14 N, 0.79 W  (37–38N for 160Ma–150Ma)  51.72 N, 1.16 W  (34–35N for 160Ma–150Ma) | Young, 2014; Foffa *et al*., 2018 |
| *Maledictosuchus riclaensis* | *Erymnoceras coronatum* ammonite Biozone, Ágreda Formation.  Middle Callovian. | ‘‘Barranco de la Paridera’’,  Ricla, Zaragoza, Spain. | 41.5° N, 1.4° W  (25–26N for 170Ma–160Ma) | Parrilla-Bel *et al*., 2013 |
| *Tyrannoneustes* cf. *lythrodectikos*, “Teleosauridae indet. teeth and fragmentary remains of *Steneosaurus* sp. and *Metriorhynchus* sp.” | Ornatenton Formation.  Middle Callovian. | Masloh quarry near the Lutternsche Egge hill in the Wiehengebirge, Germany  + Wallücke | ~52.1 N 8.3 E  (35–36N for 170Ma–160Ma) | Michelis *et al*., 1996; Waskow *et al*., 2018 [NB taxonomic ID of *T.* cf. *lythrodectikos* requires verification] |
| cf. *Tyrannoneustes*  ‘*Dakosaurus’* | Callovian-Oxfordian aged deposits that yield isolated tooth crowns | Numerous localities in Poland. Spanning north-to-south: Wapienno, Inowrocław, and  Ogrodzieniec | 52.5 N, 17.5 E  (38–38N for 160Ma–150Ma)  50.2 N, 19.3 E  (37N for 160Ma) | Young *et al*., 2013a |
| Metriorhynchidae indet. or *Dakosaurus maximus* | Kimmeridgian aged deposits | Czarnogłowy. Poland (museum specimens labelled Zarnglaff are from this locality) | 53.4 N, 14.5 E  (39–39N for 160Ma–150Ma) | Hoffmann, 2005;  Hoffmann & Bickelmann, 2008 |
| *Geosaurus* sp.  *Torvoneustes coryphaeus*  *Dakosaurus maximus*  Geosaurinae gen. et sp. (OUMNH J.1583)  ‘English rostrum’ (‘E’-clade)  *Plesiosuchus manselii*  *Torvoneustes carpenteri*  *‘Metriorhynchus’ palpebrosus* | Kimmeridge Clay Formation.  Early Kimmeridgian to early Tithonian (154-148 Ma) | Numerous localities across England,  UK. Spanning from Ely in the north, to Kimmeridge in the south | 52.2 N 0.1 E  (35–36N for 160Ma–150Ma)  50.6 N 2.1 W  (33–34N for 160Ma–150Ma) | Young *et al*., 2012, 2013b, 2014a, 2020b |
| *Dakosaurus maximus*  *‘Metriorhynchus’* cf. *palpebrosus*  *‘Metriorhynchus’* cf. *hastifer* (‘E’-clade)  *Gracilineustes acutus* | Marnes de Bléville and  Argiles d’Octeville Formations.  Kimmeridgian. | Numerous localities across Normandy, France. Most near Octeville-sur-Mer and Cap de la Hève | 49.3 N 0.1 E  (32–33N for 160Ma–150Ma) | Lepage *et al*., 2008; Young *et al*., 2012, 2020a |
| ‘Swiss rostrum’ (‘E’-clade) | Villigen Formation.  Lower Kimmeridgian. | Near Oberbuchsiten, Canton Solothurn, Switzerland | 47.1 N, 7.4 E  (NA) | Young *et al*., 2020a |
| Druegendorf ‘E’-clade geosaurine | Feuerstein and Wohlgeschichtete Kalke Formations.  Kimmeridgian. | Braunenberg, Baden-Württemberg and Drügendorf, Bavaria, Germany | 49.5 N, 6.5 E  (33–34N for 160Ma–150Ma)  49.8 N, 11.1 E (34–35N for 160Ma–150Ma) | Abel *et al*., 2020 |
| *Cricosaurus* *bambergensis*  *Cricosaurus albersdorferi*  *Cricosaurus suevicus*  *Cricosaurus elegans*  *Cricosaurus rauhuti*  *Rhacheosaurus gracilis*  *Dakosaurus maximus*  *Geosaurus giganteus*  *Geosaurus grandis* | Numerous formations, deposited in lagoonal or very shallow ecosystems (late Kimmeridgian to early Tithonian) | Numerous localities across the Franconian Alb, Germany. Most are from the Solnhofen-Daiting region. Spanning from Wattendorf in the north to Nusplingen in the south. | 50.0 N 11.1 E  (35N for 150Ma)  48.1 N 8.5 E  (33N for 150Ma) | Andrade *et al*., 2010; Young *et al*., 2012; Sachs *et al*., 2019, 2021; Herrera *et* *al*., 2021b |
| Swiss rhacheosaurin | Twannbach Formation.  Lower Tithonian | Near Evilard, Canton Bern, Switzerland | 47.1 N, 7.1 E  (NA) | Young *et al*., 2021 |
| cf. *Plesiosuchus manselii* | Tereñes Formation.  Kimmeridgian. | La Griega Beach, Asturias, Northern Spain | 43.5 N, 5.2 E  (29–29N for 160Ma–150Ma) | Young *et al*., 2012 |
| cf. *Plesiosuchus* | *Semiradiata* Biozone, middle Tithonian | Kyjov, Slovakia | 49.1 N, 20.5 E  (NA) | Čerňanský *et al*., 2019 |
| *Neustosaurus gigondarum* (nomen dubium) | Lower Valanginian | Gigondas, Département du Vaucluse, France | 44.1 N, 5.0 E  (26–29N for 140Ma–130Ma) | Raspail, 1842 |
| ‘*Cricosaurus*’ *macrospondylus* referred specimen | Lower Valanginian | Barret-le-Bas, Hautes-Alpes, France | 44.1 N, 5.4 E (NA) | Hua *et al*., 2000 |
| Plesiosuchina indet. | Lower Upper Valanginian | Escaouprès valley, Département des Bouches-du- Rhône, France | 43.3 N, 5.5 E  (NA) | Young *et al*., 2014b |
| *Geosaurus lapparenti* | Upper Valanginian | La Martre, Département du Var, France | 43.4 N, 6.3 E  (NA) | Debelmas & Strannoloubsky, 1956 |
| *Cricosaurus schroederi* | Lower Valanginian | Sachsenhagen, Lower Saxony, Germany | 52.2 N, 9.1E  (34–37N for 140Ma–130Ma) | Karl *et al*., 2006; Sachs *et al*., 2020 |
| *Enaliosuchus macrospondylus* (nomen dubium) | Lower upper Valanginian | Near Osterwald, Lower Saxony, Germany | 52.1 N, 9.3 E  (34–37N for 140Ma–130Ma) | Koken, 1883; Sachs *et al*., 2020 |
| Plesiosuchina? indet.  *Torvoneustes*? sp. | Late Valanginian | Blücher quarries at Štramberk area, Czech Republic | 49.59 N, 18.12 E  (32–35 N for 140–130 Ma) | Madzia *et al*., 2021 |
|  | | | | |
| cf. Plesiosuchina | Hybla Formation.  Lower-most Aptian | Rocca Chi Parra quarry, Montagna Grande area, Sicily, Italy | 37.909 N, 12.762 E  (NA) | Chiarenza *et al*., 2015; Sachs *et al*., 2020 |

**Supplementary References**

**Abel P, Sachs S, Young MT. 2020.** Metriorhynchid crocodylomorphs from the lower Kimmeridgian of southern Germany: evidence for a new large-bodied geosaurin lineage in Europe. *Alcheringa* **44(2)**: 312–326.

**Aiglstorfer M, Havlik P, Herrera Y. 2020.** The first metriorhynchoid crocodyliform from the Aalenian (Middle Jurassic) of Germany, with implications for the evolution of Metriorhynchoidea. *Zoological Journal of the Linnean Society* **188**: 522–551.

**Andrade MB, Young MT, Desojo JB, Brusatte SL. 2010**. The evolution of extreme hypercarnivory in Metriorhynchidae (Mesoeucrocodylia: Thalattosuchia) based on evidence from microscopic denticle morphology. *Journal of Vertebrate Paleontology* **30**: 1451–1465.

**Andrews CW. 1913**. A descriptive catalogue of the marine reptiles of the Oxford Clay, Part Two. London: British Museum (Natural History).

**Barrientos-Lara JI, Herrera Y, Fernández MS, Alvarado-Ortega J. 2016.** Occurrence of *Torvoneustes* (Crocodylomorpha, Metriorhynchidae) in marine Jurassic deposits of Oaxaca, Mexico. *Revista Brasileira de Paleontologia* **19**:415–424.

**Barrientos-Lara JI, Alvarado-Ortega J, Fernández MS. 2018.** The Marine Crocodile *Maledictosuchus* (Thalattosuchia, Metriorhynchidae) from the Kimmeridgian Deposits of Tlaxiaco, Oaxaca, Southern Mexico. *Journal of Vertebrate Paleontology* **38**(4): (1)–(14).

**Benton MJ, Taylor MA. 1984.** Marine reptiles from the Upper Lias (Lower Toarcian, Lower Jurassic) of the Yorkshire coast. *Proceedings of the Yorkshire Geological Society* **44**: 399–429.

**Brignon A. 2018.** *La collection de vertébrés jurassiques du Calvados de Pierre Tesson (1797-1874)*. Published by the author, Bourg-la-Reine, vi + 82 pp.

**Buchy M-C. 2008a.** Reevaluation of the holotype of *Plesiosaurus* (*Polyptychodon*) *mexicanus* Wieland, 1910 from the ?Upper Jurassic of Mexico: a thalattosuchian, not a sauropterygian. *Revista Mexicana de Ciencias Geologicas* **25**:517–522.

**Buchy M-C. 2008b.** New occurrence of the genus *Dakosaurus* (Reptilia, Thalattosuchia) in the Upper Jurassic of north-eastern Mexico with comments upon skull architecture of *Dakosaurus* and *Geosaurus*. *Neues Jahrbuch für Geologie und Paläontologie*, **249(1)**: 1–8.

**Buchy M-C, Vignaud P, Frey E, Stinnesbeck W, Gonzalez AH. 2006.** A new thalattosuchian crocodyliform from the Tithonian (Upper Jurassic) of northeastern Mexico. *Comptes Rendus Palevol* **5**:785–794.

**Buchy M-C, Stinnesbeck W, Frey E, Gonzalez AH. 2007.** Premiere mention du genre *Dakosaurus* (Crocodyliformes, Thalattosuchia) dans le Jurassique superieur du Mexique. *Bulletin de la Societe geologique de France* **178**: 63–69.

**Buchy M-C, Young MT, Andrade MB. 2013.** A new specimen of Cricosaurus saltillensis (Crocodylomorpha: Metriorhynchidae) from the Upper Jurassic of Mexico: evidence for craniofacial convergence within Metriorhynchidae. *Oryctos* **10**: 9–21.

**Buffetaut E. 1979.** Jurassic marine crocodilians (Mesosuchia: Teleosauridae) from central Oregon: ﬁrst record in North America. Journal of Paleontology **53**: 210–215.

**Cadena-Rangel J. 2015.** Presencia de *Cricosaurus vignaudi* (Crocodylomorpha: Familia Metriorhinchidae) en el Estado de Guerrero, Jurasico Medio. B.Sc. thesis, Universidad Nacional Autonoma de Mexico, Mexico City, Mexico, 59 pp.

**Cau A, Fanti F. 2011.** The oldest known metriorhynchid crocodylian from the Middle Jurassic of North-eastern Italy: *Neptunidraco ammoniticus* gen. et sp. nov. *Gondwana Research* **19**: 550–565.

**Čerňanský A, Schlögl J, Mlynský T, Józsa S. 2019.** First evidence of the Jurassic thalattosuchian (both teleosaurid and metriorhynchid) crocodylomorphs from Slovakia (Western Carpathians). *Historical Biology* **31(8)**: 1008–1015.

**Chiarenza A, Foffa D, Young MT, Insacco G, Cau A, Carnevale G, Catanzariti R. 2015.** The youngest record of metriorhynchid crocodylomorphs, with implications for the extinction of Thalattosuchia. *Cretaceous Research* **56**:608–616.

**Collot L. 1905**. Reptile jurassique (*Teleidosaurus gaudryi*) trouvé à Saint-Seine-l’Abbaye (Côte-d’Or). *Mémoires de l’Académie des Sciences, Arts et Belles-Lettres de Dijon* **10**: 41–45.

**Debelmas J, Strannoloubsky A. 1956**. Decouverte d’un crocodilien dans le Neocomien de La Martre (Var) *Dacosaurus lapparenti* n. sp. *Travaux Laboratoire de Geologie de l’universite de Grenoble* **33**: 89–99.

**Duffin CJ. 1979.** *Pelagosaurus* (Mesosuchia, Crocodilia) from the English Toarcian (Lower Jurassic). *Neues Jahrbuch für Geologie und Paläontologie, Monatshefte* **1979**: 475–485.

**Eudes-Deslongchamps JA. 1864.** Mémoire sur les Téléosauriens de l’époque Jurassique du Calvados. Mémoires de la Société Linnéenne de Normandie **13**: 1–138.

**Eudes-Deslongchamps JA. 1866.** Description d’une espèce inédite de Téléosaure des environs de Caen, Teleosaurus calvadosii. *Bulletin de la Société Linnéenne de Normandie* **10**: 193–223.

**Eudes-Deslongchamps E. 1867–69.** Prodrome des téléosauriens du Calvados. In: *Notes paléontologiques.* Caen & Savy: Le Blanc-Hardel, 95–162 (year 1867); 163–196 (year 1868); 197–354, 374–379, 385–392 (year 1869); pl. 10–24.

**Fernández MS, Carabajal AP, Gasparini Z, Chong Díaz G. 2011.** A metriorhynchid crocodyliform braincase from northern Chile. *Journal of Vertebrate Paleontology* **31**: 369–377.

**Fernandez MS, Herrera Y, Vennari VV, Campos L, de la Fuente M, Talevi M, Aguirre-Urreta B. 2019.** Marine reptiles from the Jurassic/Cretaceous transition at the High Andes, Mendoza, Argentina. *Journal of South American Earth Sciences* **92**: 658–673.

**Ferreira OV. 1959.** Nota sobre a presence do género *Pelagosaurus* no Lias do Tomar. *Anais da Faculdade de Ciências da Universidade do Porto* **41**: 121–125.

**Foffa D, Young MT, Brusatte SL, Graham MR, Steel L. 2018.** A new metriorhynchid crocodylomorph from the Oxford Clay Formation (Middle Jurassic) of England, with implications for the origin and diversification of Geosaurini. *Journal of Systematic Palaeontology* **16** (13):1123–1143.

**Frey E, Buchy M-C, Stinnesbeck W, Lopez-Oliva JG. 2002.** *Geosaurus vignaudi* n. sp. (Crocodyliformes: Thalattosuchia), first evidence of metriorhynchid crocodilians in the Late Jurassic (Tithonian) of central-east Mexico (State of Puebla). *Canadian Journal of Earth Sciences* **39**: 1467–1483.

**Gasparini ZB. 1980**. Un nuevo cocodrilo marino (Crocodylia, Metriorhynchidae) del Caloviano del norte de Chile. *Ameghiniana* **17**: 97–103.

**Gasparini, Z. 1985.** Los reptiles marinos jurásicos de América del Sur. *Ameghiniana* 22: 23–34.

**Gasparini Z. 1992.** Marine reptiles from the circum-Pacific region. 361–364 *In*: G. E. G. Westermann (Ed.), The Jurassic of the circum Pacific. World and regional Geology 3, Cambridge: Cambridge University Press.

**Gasparini Z, Chong-Díaz G. 1977.** *Metriorhynchus casamiquelai* n. sp. (Crocodilia, Thalattosuchia) a marine crocodile from the Jurassic (Callovian) of Chile, South America. *Neues Jahrbuch für Geologie und Paläontologie, Abhandlungen* **153**: 341–360.

**Gasparini Z, Dellapé D. 1976**. Un nuevo cocodrilo marino (Thalattosuchia, Metriorhynchidae) de la Formación Vaca Muerta (Tithoniano) de la Provincia de Neuquén (Argentina). *1° Congreso Geológico Chileno (Santiago de Chile), Actas*: C1–C21.

**Gasparini ZB, Iturralde-Vinent M. 2001.** Metriorhynchid crocodiles (Crocodyliformes) from the Oxfordian of Western Cuba. *Neues Jahrbuch für Geologie und Paläontologie Monatshefte* **9**: 534–542.

**Gasparini ZB, Iturralde-Vinent M. 2006.** The Cuban Oxfordian herpetofauna in the Caribbean Seaway. *Neues Jahrbuch für Geologie und Paläontologie* *Abhandlungen* **240**(3): 343–371.

**Gasparini Z, Vignaud P, Chong-Díaz G. 2000**. The Jurassic Thalattosuchia (Crocodyliformes) of Chile: a paleobiogeographic approach. Bulletin Société Géologique de France 171: 657–664.

**Gasparini Z, Cichowolski M, Lazo DG. 2005.** First record of *Metriorhynchus* (Reptilia: Crocodyliformes) in the Bathonian (Middle Jurassic) of the Eastern Pacific. Journal of Paleontology **79**:801–805.

**Gasparini ZB, Pol D, Spalletti LA. 2006.** An unusual marine crocodyliform from the Jurassic–Cretaceous boundary of Patagonia. Science **311**: 70–73.

**Gasparini ZB, Paulina-Carabajal A, Chong-Díaz G. 2008.** Un nuevo espécimen de cocodrilo marino del Jurásico Medio del norte de Chile: revalidación de *Metriorhynchus westermanni* (Crocodyliformes: Metriorhynchidae). *Revista Geológica de Chile* **35**: 335–346.

**Herrera Y. 2015.** Metriorhynchidae (Crocodylomorpha: Thalattosuchia) from Upper Jurassic–Lower Cretaceous of Neuquén Basin (Argentina), with comments on the natural casts of the brain. *In:* M. Fernández y Y. Herrera (Eds.) Reptiles Extintos - Volumen en Homenaje a Zulma Gasparini. *Publicación Electrónica de la Asociación Paleontológica Argentina* **15(1)**: 159–171.

**Herrera Y, Gasparini Z, Fernández MS. 2013.** A new Patagonian species of *Cricosaurus* (Crocodyliformes, Thalattosuchia): first evidence of *Cricosaurus* in middle-upper Tithonian lithographic limestone from Gondwana. *Palaeontology* **56**: 663–678.

**Herrera Y, Gasparini Z, Fernández MS. 2015.** *Purranisaurus potens* Rusconi, an enigmatic metriorhynchid from the Late Jurassic–Early Cretaceous of the Neuquén Basin. *Journal of Vertebrate Paleontology* **35**: e904790.

**Herrera Y, Fernández MS, Vennari VV. 2021a.** *Cricosaurus* (Thalattosuchia, Metriorhynchidae) survival across the J/K boundary in the High Andes (Mendoza Province, Argentina). *Cretaceous Research* **118**:104673.

**Herrera Y, Aiglstorfer M, Bronzati M. 2021b.** A new species of *Cricosaurus* (Thalattosuchia: Crocodylomorpha) from southern Germany: the first three-dimensionally preserved *Cricosaurus* skull from the Solnhofen Archipelago. *Journal of Systematic Palaeontology* **19**(2):145–167.

**Hoffmann N. 2005.** Die Vertebraten des Pommerschen Malms von Czarnogłowy, Wrzosowo, Kłęby und Bardy (Oxford/Kimmeridge, Nordwest­polen). 72 pp. Institut für Geographie und Geologie der Ernst-Moritz- Arndt-Universität Greifswald, Greifswald.

**Hoffmann N, Bickelmann C. 2008.** Upper Jurassic marine reptiles from Northwest Poland. *Palherp* 2008.

**Hua S. 2020**. A new specimen of *Teleidosaurus calvadosii* (Eudes-Deslongchamps, 1866) (Crocodylia, Thalattosuchia) from the Middle Jurassic of France. *Annales de Paléontologie* **106**: 102423.

**Hua S, Vignaud P, Atrops F, Clément A. 2000.** *Enaliosuchus macrospondylus* Koken, 1883 (Crocodylia, Metriorhynchidae) du Valanginien de Barret-le-Bas (Hautes Alpes, France): un cas unique de remontée des narines externes parmi les crocodiliens. *Géobios* **33**: 467–474.

**Karl H-V, Gröning E, Brauckmann C, Knötschke N. 2006.** Revision of the genus *Enaliosuchus* Koken, 1883 (Archosauromorpha: Metriorhynchidae) from the Early Cretaceous of Northwestern Germany. *Studia Geologica Salmanticensia* **42**: 49–59.

**Koken E. 1883.** Die Reptilien der norddeutschen unteren Kreide. *Zeitschrift der Deutschen Geologischen Gesellschaft* **35**: 735–827.

**Larsson HCE, Dececchi TA, Montefeltro FC. 2012.** A new metriorhynchid (Crocodyliformes, Thalattosuchia) from the early cretaceous of Colombia (Rosa Blanco Formation, late Valanginian). *Ameghiniana (Suplemento)* **48**: 86–87.

**Lepage Y, Buffetaut E, Hua S, Martin JE, Tabouelle J. 2008.** Catalogue descriptif, anatomique, geologique et historique des fossiles présentés a l’exposition « Les Crocodiliens fossiles de Normandie » (6 novembre - 14 decembre 2008). *Bulletin de la Société Géologique de Normandie et des Amis du Muséum du Havre* **95**:5–152.

**Madzia D, Sachs S, Young MT, Lukeneder A, Skupien P. 2021.** Evidence of two lineages of metriorhynchid crocodylomorphs in the Lower Cretaceous of the Czech Republic. *Acta Palaeontologica Polonica* **66(2):** 357–367.

**Mercier J. 1933.** Contribution a l’étude des métriorhynchidés (crocodiliens). *Annales de Paleontologie* **22**: 99–119.

**Michelis I, Sander PM, Metzdorf R, Breitkreuz H. 1996.** Die Vertebratenfauna des Calloviums (Mittlerer Jura) aus dem Steinbruch Störmer (Wallücke, Wiehengebirge). *Geologie und Paläontologie in Westfalen* **44**: 66 p.

**Ősi A, Young MT, Galácz A, Rabi M. 2018.** A new large-bodied thalattosuchian crocodyliform from the Lower Jurassic (Toarcian) of Hungary, with further evidence of the mosaic acquisition of marine adaptations in Metriorhynchoidea. *PeerJ* **6**: e4668.

**Parrilla-Bel J, Young MT, Moreno-Azanza M, Canudo JI. 2013.** The first metriorhynchid crocodylomorph from the Middle Jurassic of Spain, with implications for evolution of the subclade Rhacheosaurini. *PLoS One* **8**: e54275.

**Pierce SE, Benton MJ. 2006.** *Pelagosaurus typus* Bronn, 1841 (Mesoeucrocodylia: Thalattosuchia) from the Upper Lias (Toarcian, Lower Jurassic) of Somerset, England. *Journal of Vertebrate Paleontology* **26(3)**: 621–635.

**Pol D, Gasparini Z. 2009.** Skull anatomy of *Dakosaurus andiniensis* (Thalattosuchia: Crocodylomorpha) and the phylogenetic position of Thalattosuchia. *Journal of Systematic Palaeontology* **7**: 163–197.

**Raspail E. 1842.** Observations sur un nouveau genre de Saurien fossile, le *Neustosaurus* *Gigondarum*, N. avec quelques notes geologiques sur la commune de Gigondas. Raspail, Paris, p. 56.

**Riou B, Bennourine M, Klee N, Lutz M. 2021.** Jurassic marine crocodiles in the Monts d’Ardèche UNESCO Global Geopark. *Geoconservation Research* **4(2):** 368–377.

**Sachs S, Young MT, Abel P, Mallison H. 2019.** A new species of *Cricosaurus* (Thalattosuchia, Metriorhynchidae) from the Upper Jurassic of southern Germany. *Acta Palaeontologica Polonica* **64**: 345–356.

**Sachs S, Young MT, Hornung J. 2020.** The enigma of *Enaliosuchus*, and a reassessment of the Early Cretaceous fossil record of Metriorhynchidae. *Cretaceous Research* **114**: 104479.

**Sachs S, Young MT, Abel P, Mallison H. 2021.** A new species of *Cricosaurus* (Thalattosuchia, Metriorhynchidae) based upon a remarkably well-preserved skeleton from the Upper Jurassic of Germany. *Palaeontologia Electronica* **24(2)**: a24

**Soto-Acuña S, Otero RA, Rubilar-Rogers D, Vargas AO. 2015a.** Arcosaurios no avianos de Chile. *Publicación Ocasional del Museo Nacional de Historia Natural, Chile,* **63**: 209–263.

**Soto-Acuña S, Otero RA, Alarcón J, Ossa-Fuentes L, Rojas O. 2015b.** Presencia de crocodrilos marinos (Thalattosuchia: Metriorhynchidae) en la Formación Cerritos Bayos 9Oxfordiano0, Cuenca de Tarapacá, Región de Antofagasta. 690–693 *In*: Charrier R, Hervé F, Maksaev V, Mpodozis C and Oyarzún J (eds.), Actas del XIV Congreso Geológico de Chile, Vol. III. October 4-8, 2015, La Serena, Chile.

**Soto-Acuña SY, Otero RA. 2014.** Overview of the fossil Crocodyliformes (Archosauria: Pseudosuchia) from Chile. En: Actas del IV Simposio Paleontología en Chile. Valdivia: 56.

**Steel R. 1973.** Crocodylia. Handbuch der Paläoherpetologie, Teil 16. Stuttgart: Gustav Fischer Verlag. 116 p.

**Telles Antunes M. 1967.** Um Mesosuquiano do Liasico de Tomar (Portugal). *Memorias dos Serviços Geologicos de Portugal new serie* **13**: 66.

**Vignaud P, Gasparini Z. 1996.** New *Dakosaurus* (Crocodylomorpha, Thalattosuchia) from the Upper Jurassic of Argentina. *Comptes Rendus de l’Académie des Sciences, Paris 2* **322**: 245–250.

**Waskow K, Grzegorczyk D, Sander PM**. 2018. The first record of *Tyrannoneustes* (Thalattosuchia: Metriorhynchidae): a complete skull from the Callovian (late Middle Jurassic) of Germany. *PalZ* **92**: 457–480.

**Westphal F. 1961.** Zur Systematik der deutschen und englischen Lias Krokodilier. *Neues Jahrbuch für Geologie und Paläontologie, Abhandlungen* **113**: 207–217.

**Westphal F. 1962.** Die Krokodilen des deutschen und englischen oberen Lias. *Palaeontographica, Abteilung A* **118**: 1–96.

**Wieland GR. 1910.** *Plesiosaurus* (*Polyptychodon*) *mexicanus* Wieland. *Parergones del Instituto Geologico de Mexico* **3**: 359–365.

**Wilberg EW. 2015**. A new metriorhynchoid (Crocodylomorpha, Thalattosuchia) from the Middle Jurassic of Oregon and the evolutionary timing of marine adaptations in thalattosuchian crocodylomorphs. *Journal of Vertebrate Paleontology* **35**: e902846.

**Young MT. 2014.** Filling the ‘Corallian Gap’: re-description of a metriorhynchid crocodylomorph from the Oxfordian (Late Jurassic) of Headington, England. *Historical Biology* **26(1)**: 80–90.

**Young MT, Andrade MB. 2009.** What is *Geosaurus*? Redescription of *G. giganteus* (Thalattosuchia: Metriorhynchidae) from the Upper Jurassic of Bayern, Germany. *Zoological Journal of the Linnean Society* **157**: 551–585.

**Young MT, Andrade MB, Brusatte SL, Sakamoto M, Liston J. 2013a**. The oldest known metriorhynchid super-predator: a new genus and species from the Middle Jurassic of England, with implications for serration and mandibular evolution in predacious clades. *Journal of Systematic Palaeontology* 4: 475–513.

**Young MT, Andrade MB, Cornée J-J, Steel L, Foffa D. 2014b.** Re-description of a putative Early Cretaceous “teleosaurid” from France, with implications for the survival of metriorhynchids and teleosaurids across the Jurassic-Cretaceous Boundary. *Annales de Paléontologie* **100**: 165–174.

**Young MT, Andrade MB, Etches S, Beatty BL. 2013b.** A new metriorhynchid crocodylomorph from the Lower Kimmeridge Clay Formation (Late Jurassic) of England, with implications for the evolution of dermatocranium ornamentation in Geosaurini. *Zoological Journal of the Linnean Society* 169(4): 820–848.

**Young MT, Brignon A, Sachs S, Hornung JJ, Foffa D, Kitson JJN, Johnson MM, Steel L. 2021.** Cutting the Gordian knot: a historical and taxonomic revision of the Jurassic crocodylomorph *Metriorhynchus*. *Zoological Journal of the Linnean Society* **192(2)**: 510–553.

**Young MT, Brusatte SL, Ruta M, Andrade MB. 2010.** The evolution of Metriorhynchoidea (Mesoeucrocodylia: Thalattosuchia): an integrated approach using geometrics morphometrics, analysis of disparity and biomechanics. *Zoological Journal of the Linnean Society* **158**: 801–859.

**Young MT, Brusatte SL, Andrade MB, Desojo JB, Beatty BL, Steel L, Fernández MS, Sakamoto M, Ruiz-Omeñaca JI, Schoch RR. 2012.** The cranial osteology and feeding ecology of the metriorhynchid crocodylomorph genera *Dakosaurus* and *Plesiosuchus* from the Late Jurassic of Europe. *PLoS One* **7**: e44985.

**Young MT, Foffa D, Steel L, Etches S. 2020b.** Macroevolutionary trends in the genus *Torvoneustes* (Crocodylomorpha: Metriorhynchidae) and discovery of a giant specimen from the Late Jurassic of Kimmeridge, UK. *Zoological Journal of the Linnean Society* **189**: 483–493.

**Young MT, Sachs S, Abel P, Foffa D, Herrera Y, James Kitson JN. 2020a.** Convergent evolution and possible constraint in the posterodorsal retraction of the external nares in pelagic crocodylomorphs. *Zoological Journal of the Linnean Society* **189**: 494–520.

**Young MT, Steel L. 2019.** Chapter 19: Reptiles – Crocodylomorphs. In: Lord AR. (eds.) *Fossils from the Lias of the Yorkshire Coast*. Palaeontological Association Field Guide 15. 403 pp.

**Young MT, Steel L, Middleton H. 2014a.** Evidence of the metriorhynchid crocodylomorph genus Geosaurus in the Lower Kimmeridge Clay Formation (Late Jurassic) of England. *Historical Biology* **26(5)**: 551–555.
