# Supplementary material for: Thalattosuchian crocodylomorphs from European Russia, and new insights into metriorhynchid tooth serration evolution and their palaeolatitudinal distribution
Source: PeerJ. 2023 Aug 11;11:e15781. doi: 10.7717/peerj.15781 (PMC10424675; doi:10.7717/peerj.15781)
Supplement: Supplemental Information 2 [file peerj-11-15781-s002.pdf]

SUPPLEMENTARY FIGURES

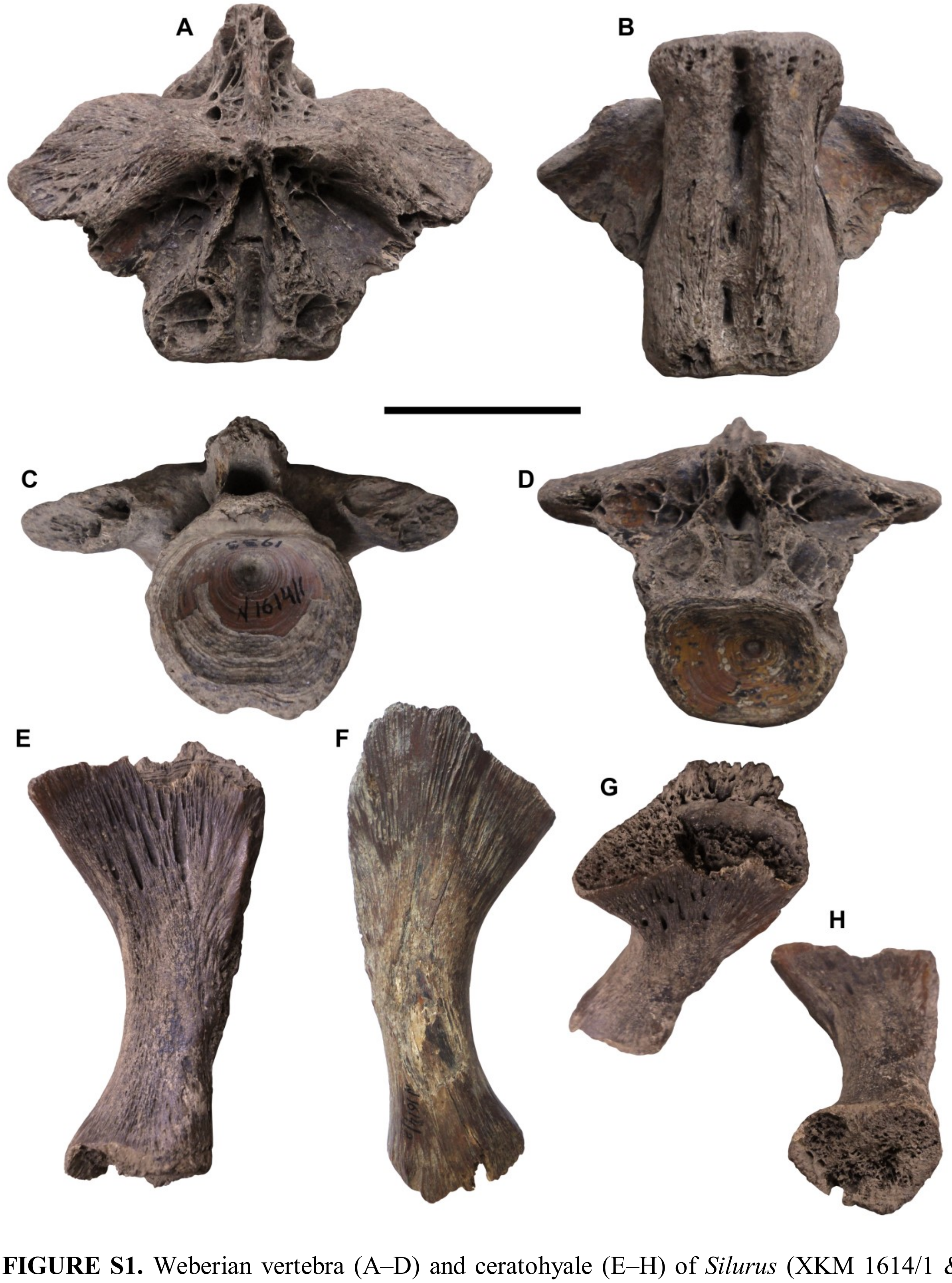

FIGURE S1. Weberian vertebra (A–D) and ceratohyale (E–H) of *Silurus* (XKM 1614/1 & 1614/2**)** from Quaternary deposits of Khoroshovsky Island on the Volga River near Khvalynsk, Saratov Oblast, Russia.

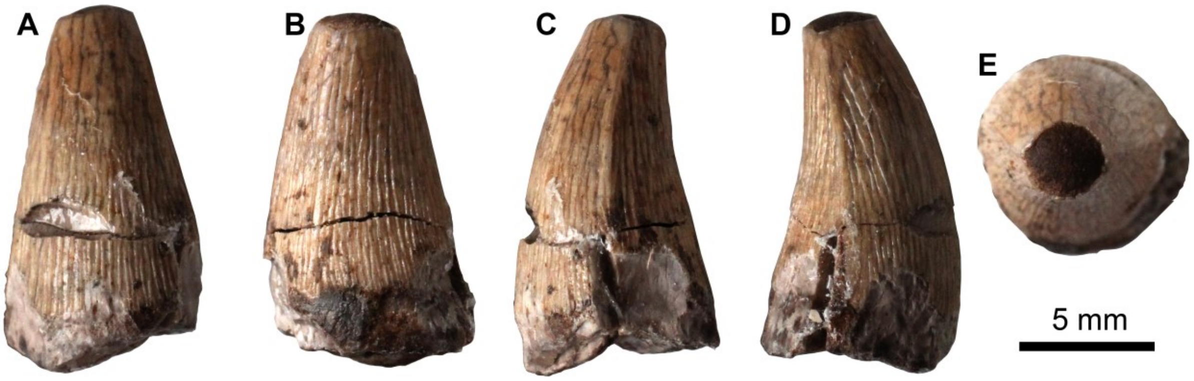

FIGURE S2. Thalattosuchia indet. tooth crown (PIN 5819/5) from the lower Bajocian of Tonkiy Gully, Volgograd Oblast, Russia; in labial (A), lingual (B), mesial/distal (C, D), and apical (E) views.

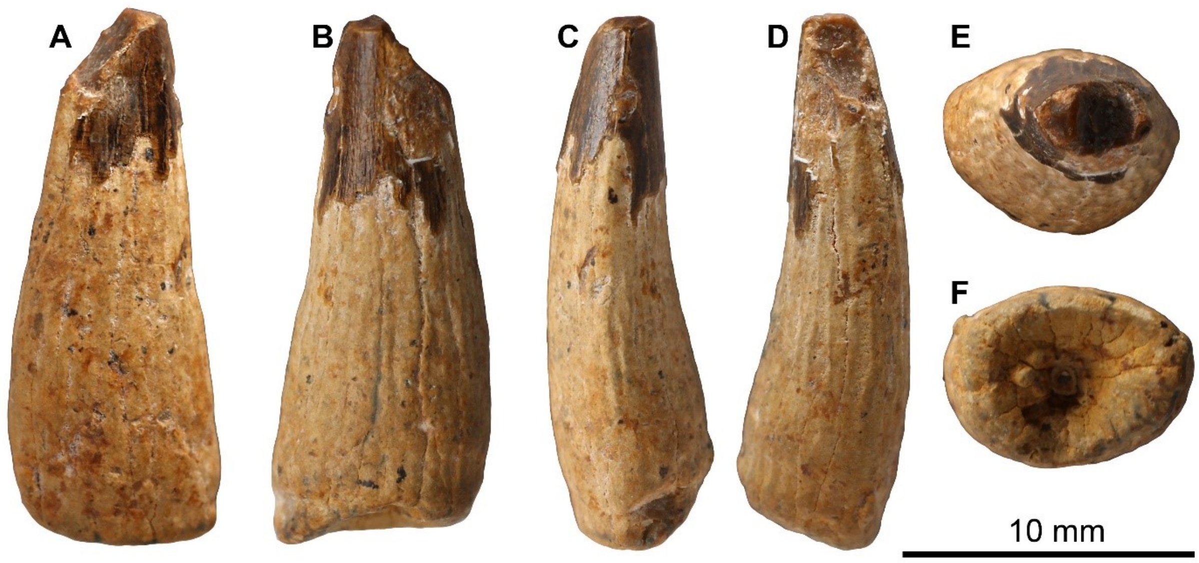

FIGURE S3. Metriorhynchidae indet. (PIN 5819/7) from the middle Callovian of the Rechitsy Village, Moscow Oblast, Russia; in labial (A), lingual (B), distal (C), mesial (D), apical (E) and basal (F) views.

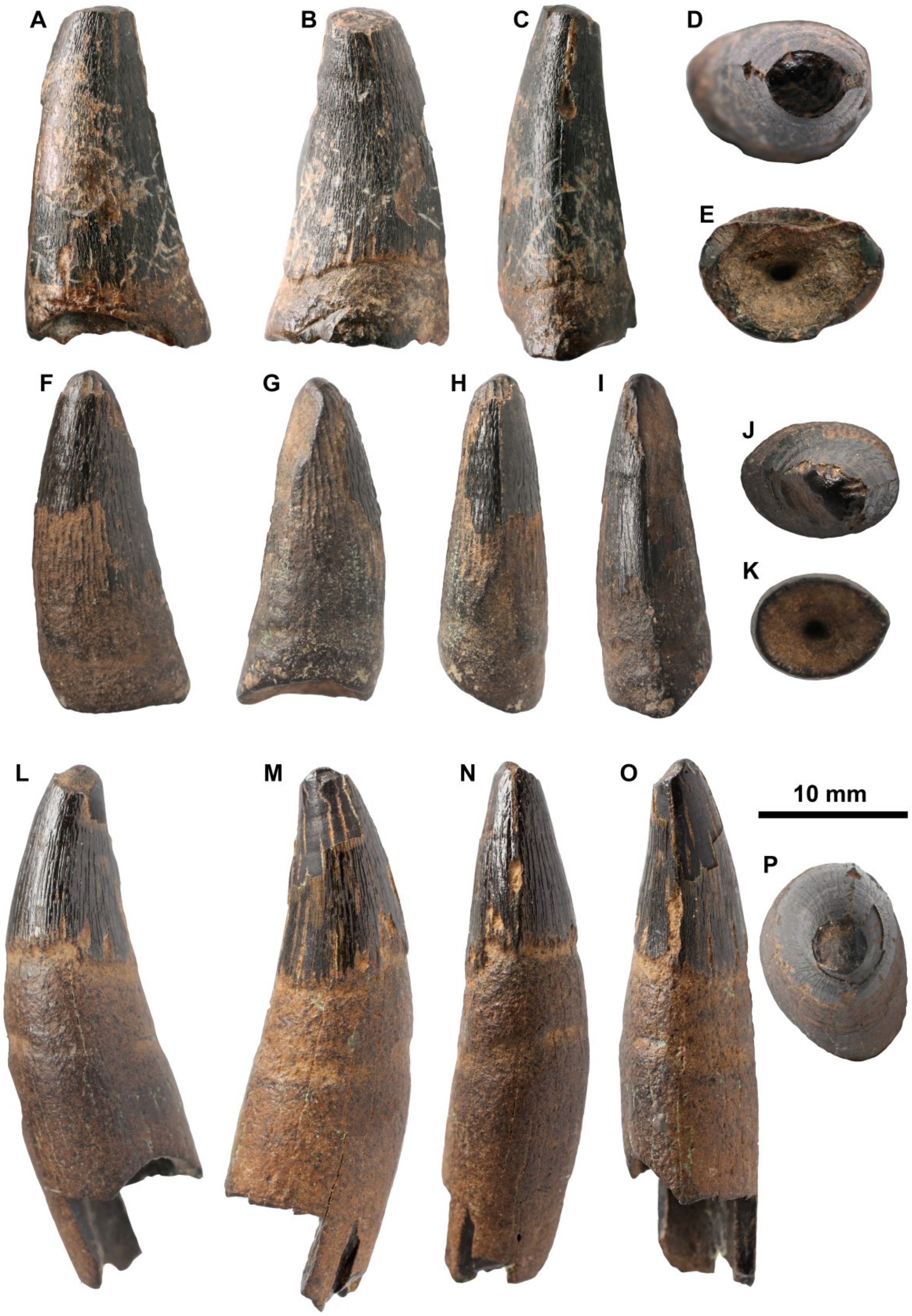

FIGURE S4. Metriorhynchid tooth crowns from the lower Callovian of the Unzha River, Kostroma Oblast, Russia. **A–E,** Metriorhynchidae indet. (PIN 5819/6). **F–K,** Metriorhynchidae indet. (PIN 5819/1). **l–P,** Metriorhynchidae indet. (PIN 5819/2). Teeth are depicted in labial (A, F, L), lingual (B, G, M), distal (C, I, O), mesial (H, N), apical (D, J, P), and basal (E, K) views.

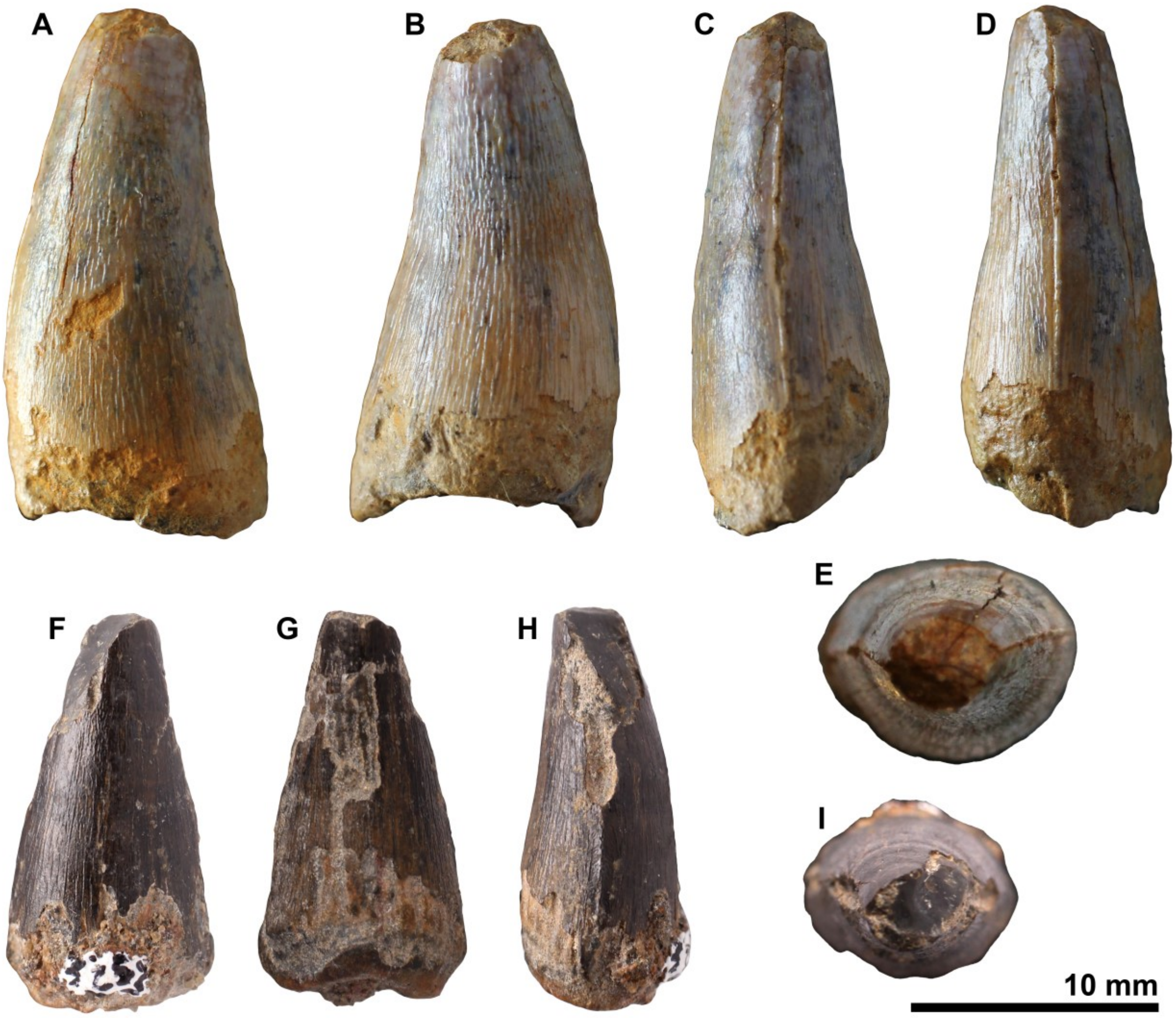

FIGURE S5. Tooth crowns of Metriorhynchidae cf. *Thalattosuchus* from the lower and middle Callovian of European Russia. **A–E,** cf. *Thalattosuchus* (MRUM 1315/1) from the lower Callovian of Gumny village, Republic of Mordovia, Russia; **F–I,** cf. *Thalattosuchus* (PIN 5477/3253) from the middle Callovian, Mikhailovcement Quarry, Ryazan Oblast, Russia. Crowns are depicted in labial (A, F), lingual (B, G), mesial (C, H), distal (D), and apical (E, I) views.

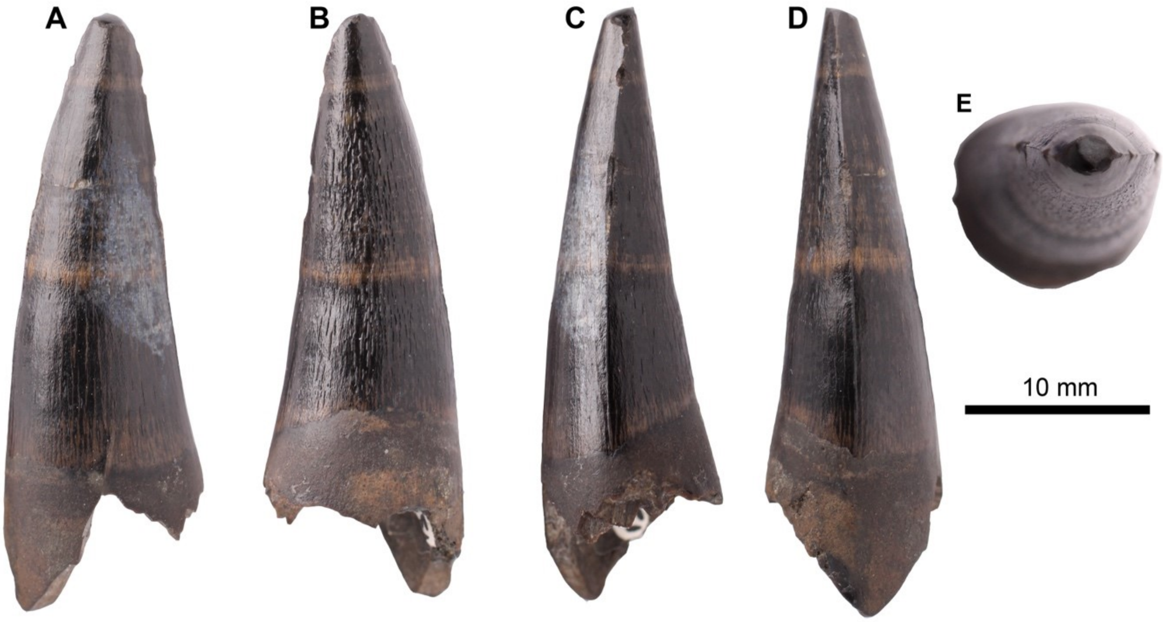

FIGURE S6. *Tyrannoneustes* sp. tooth crown (PIN 5477/2451) from the middle Callovian of Ryasan Oblast, Russia; in labial (A), lingual (B), distal (C), mesial (D), and apical (E) views.

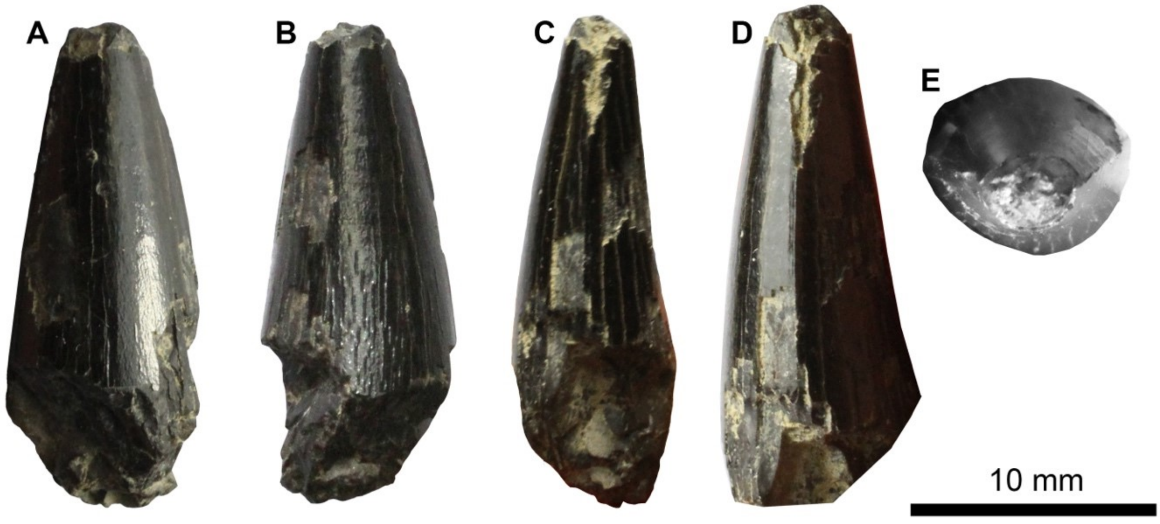

FIGURE S7. *Tyrannoneustes* sp. tooth crown (PIN 5818/9) from the middle Callovian of Moscow Oblast, Russia; in labial (A), lingual (B), mesial (C), distal (D), and apical (E) views.

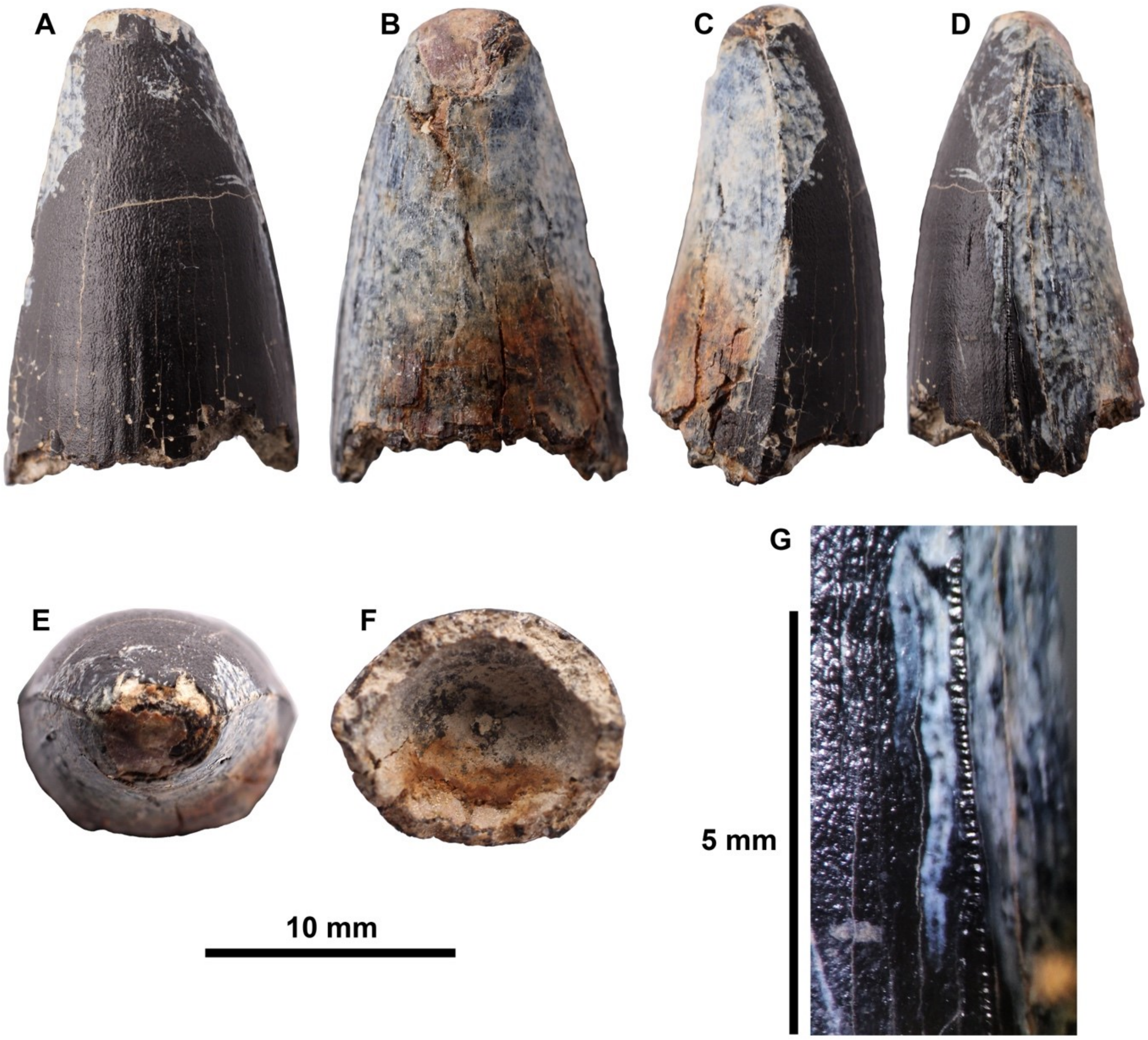

FIGURE S8. Geosaurini indet. Morphotype 1 tooth crown (SSU 14/31) from the lower Callovian of Saratov; in labial (A), lingual (B), mesial and distal (C, D), apical (E), and basal (F) views, and magnified carina (G).

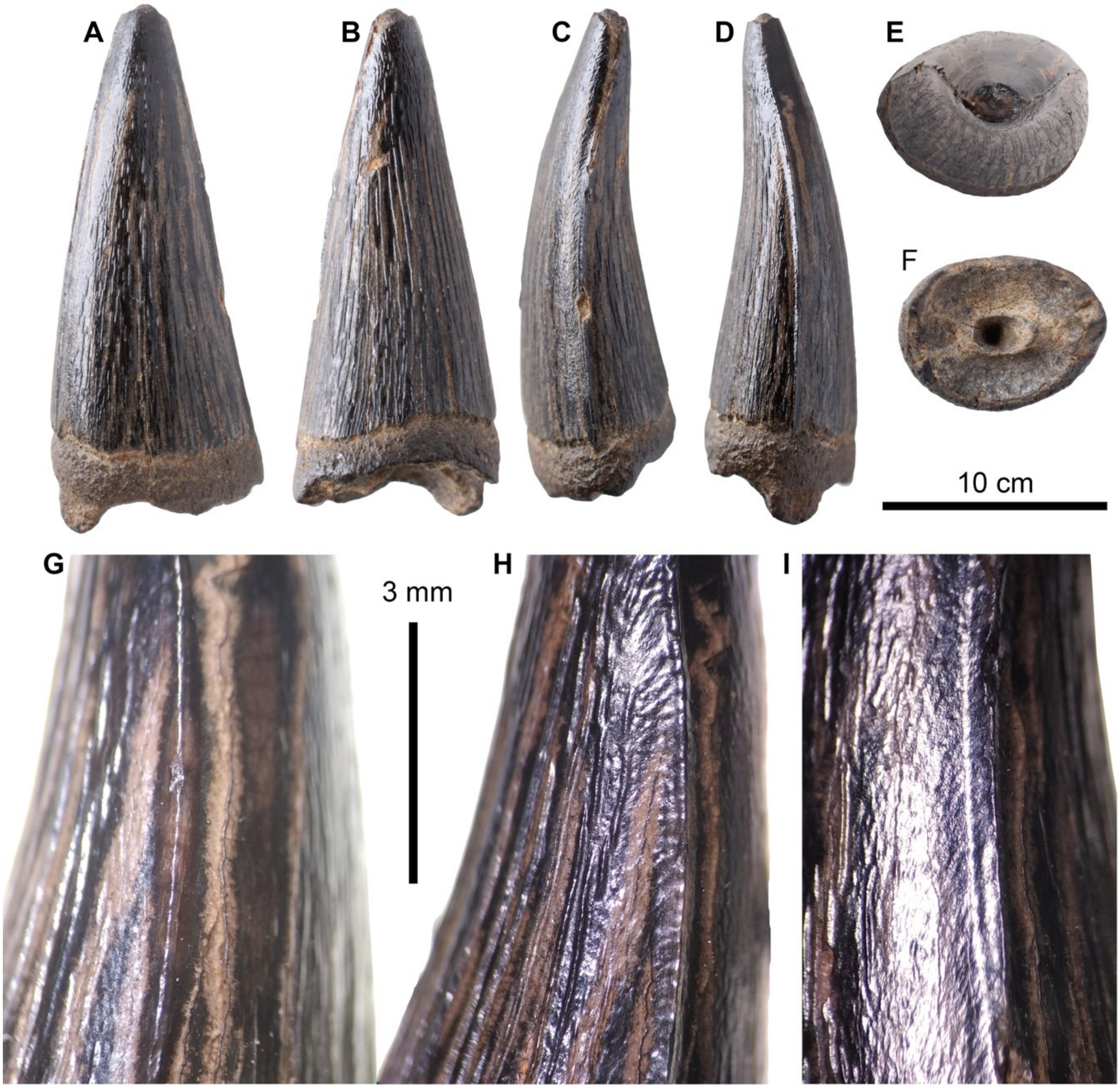

FIGURE S9. Geosaurini indet. Morphotype 2 tooth crown (SGM BX-12) from the lower Callovian of Makariev District, Kostroma Oblast; in labial (A), lingual (B), distal (C), mesial (D), apical (E), and basal (F) views; magnified photographs of the carina (G–I).

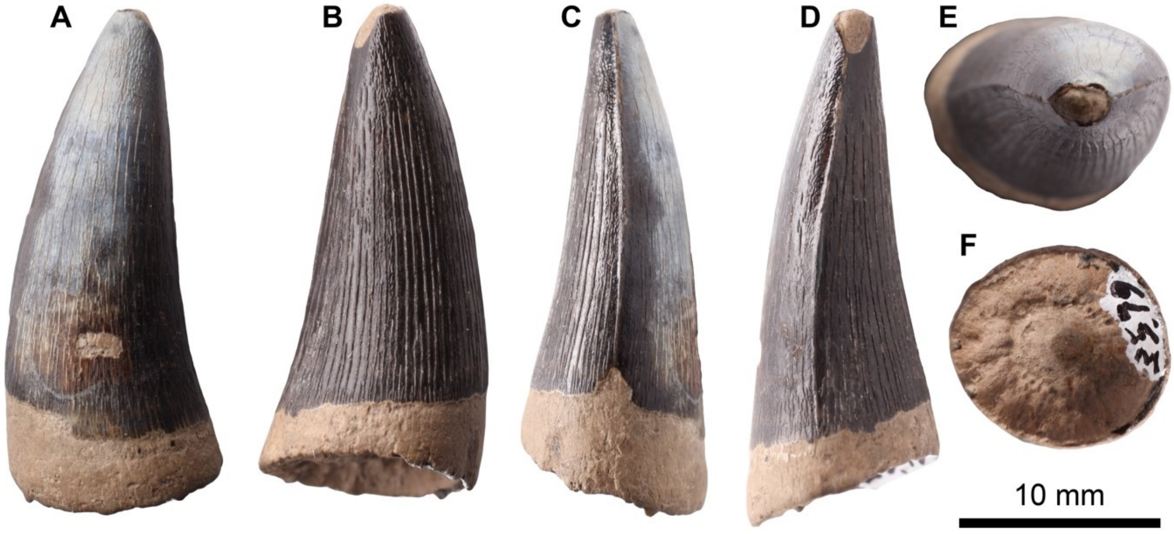

FIGURE S10. Metriorhynchidae cf. 'E'-clade tooth crown (PIN 5477/3579) from the Oxfordian–Kimmeridgian of Rybaki village, Moscow Oblast, Russia; in labial (A), lingual (B), mesial (C), distal (D), apical (E), and basal (F) views.

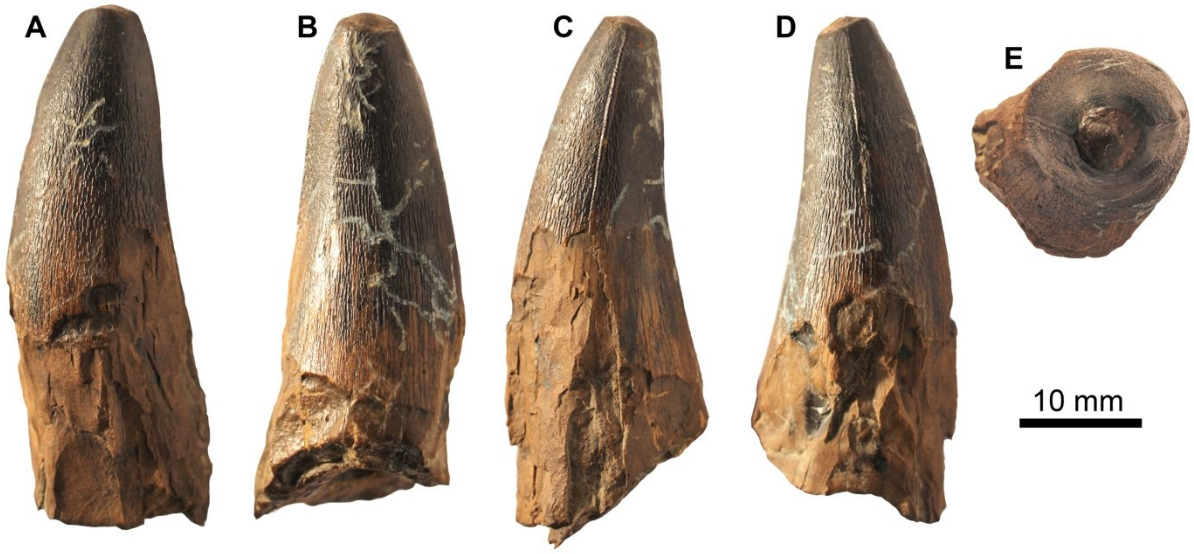

FIGURE S11. *Torvoneustes* sp. tooth crown (UPM 3026) from the Oxfordian of Okshovo village, Vladimir Oblast, Russia; in labial (A), lingual (B), distal (C), mesial (D), and apical (E) views.
